# Who Blames the Moon for Poor Sleep? An Exploratory Online Survey

**DOI:** 10.3390/clockssleep8020036

**Published:** 2026-06-22

**Authors:** Christian Cajochen

**Affiliations:** 1 Centre for Chronobiology, Psychiatric Hospital of the University of Basel, 4002 Basel, Switzerland; christian.cajochen@upk.ch; 2 Research Cluster Molecular and Cognitive Neurosciences, University of Basel, 4002 Basel, Switzerland; 3 University Centre for Sleep, Epilepsy and Circadian Medicine, University Hospital Basel, 4002 Basel, Switzerland

**Keywords:** moon, sleep, lunar phase, attribution, belief, survey, expectation

## Abstract

The belief that the moon disturbs sleep is widespread, but the factors associated with it remain poorly understood. I therefore examined how frequently poor sleep is attributed to moon phases, whether this varied across the lunar cycle, and which personal and environmental factors were associated with “moon blaming”. Data were derived from an ongoing online survey. At the time of analysis, 1815 participants had completed a 16-item questionnaire assessing sleep quality, sleep duration, sleep timing on workdays and free days, alarm clock use, environmental and personal sleep-disturbing factors, residential setting, age, gender, attention to lunar phases, and whether the moon was perceived as a cause of poor sleep. The primary outcome was endorsement of the moon as a sleep-disturbing factor. Logistic regression with stepwise Akaike information criterion selection was used to identify the strongest predictors of attributing the moon for poor sleep. Questionnaire timing was also examined across the lunar cycle. Among environmental factors, the moon was the most frequently endorsed cause of poor sleep (36%), followed by outdoor temperature (31%), indoor noise (26%), and bad weather (22%). Rumination was the most commonly reported personal factor (73%), but it did not predict moon attribution. Instead, the strongest correlates were weather-related sleep complaints, tracking lunar phases, age, and gender, with endorsement increasing with age and being more common among women. Moon-related complaints also peaked during the week after the full moon. These findings suggest that perceived lunar effects on sleep are shaped, at least in part, by attributional and expectation-related processes.

## 1. Introduction

Claims that the moon disturbs sleep are culturally prevalent but scientifically disputed. In 2013, we reported that subjective and objective measures of sleep varied with lunar phase in a controlled laboratory setting [1]. However, subsequent studies have produced conflicting results, with some reporting an association between lunar phase and sleep and others finding no clear effect [2,3,4]. This raises questions not only about whether lunar phase directly influences sleep, but also about why many people continue to believe that it does.

Poor sleep is often explained through a mixture of internal and external attributions. Some individuals emphasize rumination, stress, or irregular schedules, whereas others point to environmental influences such as noise, temperature, weather, or moonlight. The moon may occupy a special position among these explanations because it is both highly salient and culturally meaningful. It may therefore act not only as a possible environmental cue, but also as an interpretive framework for everyday sleep complaints [5,6].

Here, I addressed a complementary question to the usual lunar-sleep debate: who blames the moon for poor sleep? Specifically, I aimed to quantify how often the moon is endorsed as a sleep-disturbing factor relative to other personal and environmental influences, identify predictors of such endorsement, and test whether moon-related complaints vary according to questionnaire timing across the lunar cycle.

## 2. Results

### 2.1. Endorsement of Sleep-Disturbing Factors

**Figure 1:**
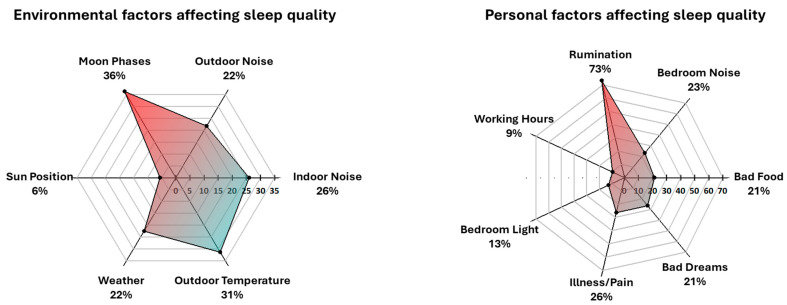
**Endorsement of sleep-disturbing factors.** (**left-hand panel**) Proportion of participants endorsing environmental factors as disturbing sleep. The moon was the most frequently endorsed environmental factor, followed by outdoor temperature, indoor noise, and bad weather. (**right-hand panel**) Proportion of participants endorsing personal factors as disturbing sleep. Rumination or worrying thoughts were the most frequently reported personal factor.

Among environmental sleep-disturbing factors, the moon was most frequently endorsed (36%), followed by outdoor temperature (31%), indoor noise (26%), and bad weather (22%) (Figure 1, left-hand panel). Among personal factors, rumination was by far the most frequently reported complaint, endorsed by 73% of participants. The five most commonly cited reasons were stress, children, nervousness, inner restlessness, and menopausal symptoms (Figure 1 right-hand panel and Figure S1).

### 2.2. Predictors of Blaming the Moon for Poor Sleep

**Figure 2:**
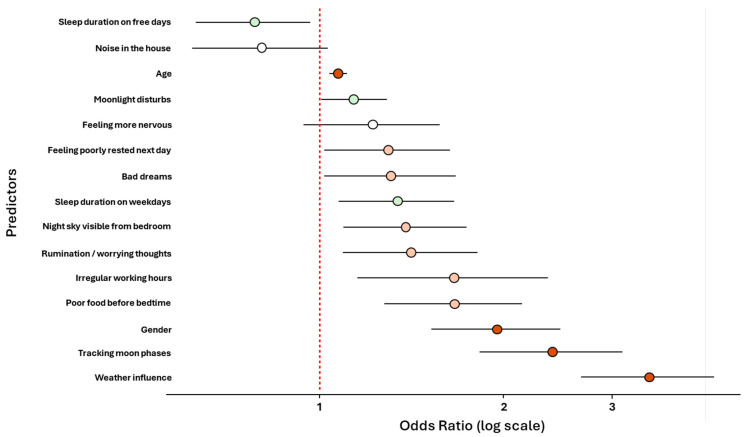
**Predictors of attributing poor sleep to the moon.** Odds ratios (OR) and 95% confidence intervals from the final multivariable logistic regression model (stepwise AIC selection). Values greater than 1 indicate a positive association with attributing poor sleep to the moon. Predictors shown include weather, tracking lunar phases, gender, and age. In red: strong/core predictors; in orange: moderate predictors and in green: small predictors, only the predictors in red were included in the final stepwise model.

Multivariable logistic regression with stepwise AIC selection identified several variables associated with attributing poor sleep to the moon (Figure 2). Endorsing weather as a sleep-disturbing factor (OR = 3.41, 95% CI [2.66, 4.39], *p* < 0.001) and tracking lunar phases (OR = 2.38, 95% CI [1.82, 3.11], *p* < 0.001) were positively associated with moon attribution.

Gender and age were also associated with the outcome: women were more likely than men to attribute poor sleep to the moon (OR = 1.93, 95% CI [1.52, 2.46], *p* < 0.001), and the likelihood of endorsement increased with age (OR = 1.07 per year, 95% CI [1.04, 1.11], *p* < 0.01).

In contrast, rumination, despite being the most frequently reported sleep complaint, was not associated with moon attribution. Overall, these findings suggest that attributing poor sleep to the moon is more closely related to environmental attribution tendencies and attention to lunar phases than to commonly reported personal sleep complaints.

### 2.3. Variation Across the Lunar Cycle

**Figure 3:**
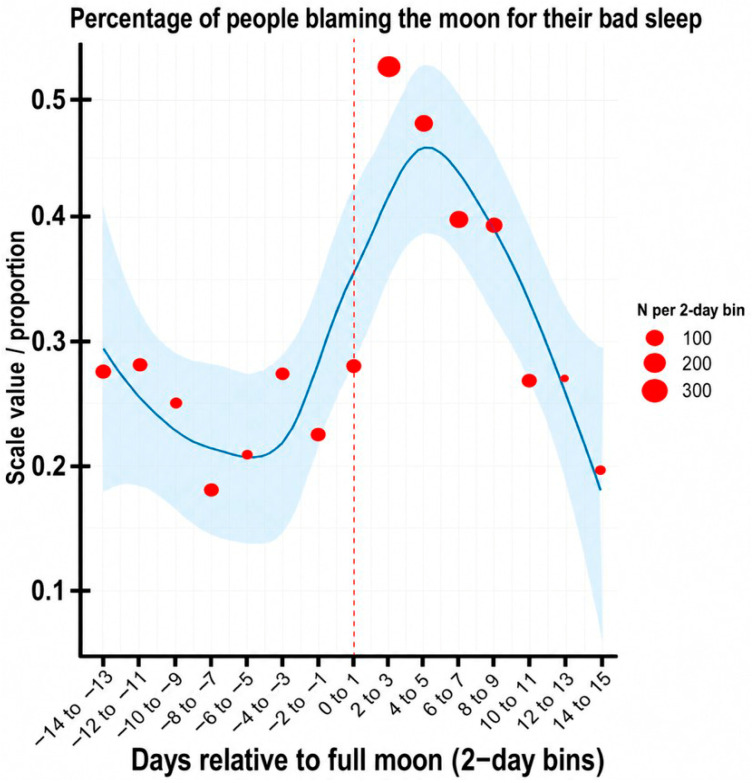
**Variation in moon attribution across the lunar cycle.** Proportion of participants attributing poor sleep to the moon as a function of days relative to the nearest full moon (±15 days), aggregated into 2-day bins. Point size reflects the number of observations per bin. The solid line represents a LOESS-smoothed trend; the dashed vertical line indicates the full moon (day 0). The light blue colour shaded uncertainty band indicates the 95% confidence interval for the estimated mean curve.

Moon attribution varied as a function of questionnaire timing across the lunar cycle (Figure 3). When responses were aligned to the nearest full moon and grouped into 2-day bins within a ±15-day window, the proportion of participants endorsing the moon showed a non-uniform temporal pattern.

Specifically, endorsement tended to increase following the full moon, reached a peak during the subsequent week, and declined thereafter. This pattern, visualized using LOESS smoothing, indicates that moon attribution varies across the lunar cycle rather than being evenly distributed over time.

## 3. Discussion

Belief that the moon disrupts sleep is widespread, yet empirical evidence for a physiological effect remains inconsistent. The present study approaches this question from a different angle: rather than testing whether the moon affects sleep, I examined which individuals are most likely to attribute poor sleep to it. By doing so, the focus from biological effects was shifted to cognitive and attributional processes.

Two findings stand out. First, attributing poor sleep to the moon was more closely linked to environmental attribution tendencies than to commonly reported sleep complaints. In the multivariable model, endorsing weather as a sleep-disturbing factor and tracking lunar phases were both strongly associated with moon attribution, whereas rumination, despite being highly prevalent, was not. This dissociation suggests that moon-related beliefs may reflect a broader explanatory style in which sleep disturbances are attributed to external, environmental causes rather than internal states.

Second, moon attribution followed a systematic temporal pattern across the lunar cycle, with higher endorsement observed in the period following the full moon. This pattern indicates that moon-related complaints are not randomly distributed over time. One interpretation is that expectations about the full moon shape retrospective reporting, increasing the likelihood that individuals notice or recall disturbed sleep during this period. Such processes could give rise to a self-reinforcing cycle of belief and experience. At the same time, the present data do not allow conclusions about underlying mechanisms, and a physiological contribution, if present, cannot be excluded.

Several limitations warrant consideration. The sample was self-selected, and all measures relied on retrospective self-report without objective sleep assessment. The analyses were exploratory and cross-sectional, and model selection was data-driven, which may limit generalizability and increase susceptibility to overfitting. In addition, the directionality of associations remains unclear; for example, it is unknown whether tracking lunar phases promotes moon attribution or reflects pre-existing beliefs. Further, in the questionnaire, education or religion/religiosity was not assessed, both of which may influence attributional processes and beliefs about lunar effects on sleep.

Despite these limitations, the findings highlight that endorsement of lunar effects on sleep is not only common but also systematically patterned. Therefore, the moon may not only function as a potential physiological influence, but also as a prominent, salient, and culturally accessible framework through which to interpret sleep disturbances.

## 4. Materials and Methods

### 4.1. Study Design and Participants

Data were derived from an ongoing online survey of 1815 respondents aged 10–89 years (69.0% women, 30.5% men, 0.5% other) who completed a 16-item questionnaire. The survey was exploratory and designed to assess subjective sleep characteristics together with perceived personal and environmental influences on sleep. The present analysis includes all fully completed responses collected between the launch of the survey on 6 February 2017 and the predefined data cut-off on 20 January 2026, which was selected for the purpose of conducting the analysis.

### 4.2. Measures

The 16-item questionnaire assessed subjective sleep quality, sleep duration, sleep timing on workdays and free days, alarm clock use, residential setting, age, gender, whether participants followed lunar phases, and whether they regarded the moon as a cause of poor sleep. Participants also indicated personal and environmental factors they believed negatively affected their sleep.

The primary outcome variable was endorsement of the moon as a sleep-disturbing factor. Candidate predictors included demographic variables, sleep-related variables, endorsement of other disturbing environmental and personal factors, and attention to lunar phases.

### 4.3. Statistical Analysis

A logistic regression to model endorsement of the moon as a sleep-disturbing factor was used. To identify the most informative set of predictors, model selection was performed using stepwise Akaike information criterion (AIC). Odds ratios and 95% confidence intervals were derived from the final model. In addition, questionnaire timing was examined across the lunar cycle to assess whether moon attribution varied as a function of days relative to the full moon.

All analyses were exploratory. Data were analyzed in R (RStudio 2026.01.0+392 “Apple Blossom” Release). Questionnaire data were imported using *readxl* and processed with *dplyr*. Incomplete responses were excluded. Selected items were reverse-coded. Age was treated as a continuous variable.

Endorsement of the moon as a sleep-disturbing factor was modeled using logistic regression (glm, binomial). Multicollinearity was assessed using correlations, variance inflation factors (*car*), and redundancy analysis (*rms*; R^2^ = 0.80). Model selection was performed using stepwise AIC (stepAIC, *MASS*).

Odds ratios and 95% confidence intervals were obtained by exponentiating model coefficients. Model performance was evaluated using accuracy and AUC (*pROC*; threshold = 0.5). Statistical significance was assessed at α = 0.05; given the exploratory nature of the analyses, p-values were interpreted descriptively.

To assess variation across the lunar cycle, questionnaire dates were aligned to the nearest full moon and expressed as days relative to it (±15 days). These were grouped into 2-day bins, and the proportion of participants endorsing the moon as a sleep-disturbing factor was calculated per bin. Trends were visualized using LOESS smoothing, with point size reflecting the number of observations.

## 5. Conclusions

Attributing poor sleep to the moon is common and is more strongly associated with environmental attribution tendencies, attention to lunar phases, age, and gender than with prevalent subjective complaints such as rumination. Moon-related complaints also show a temporal pattern, peaking in the period following the full moon. Together, these findings suggest that perceived lunar effects on sleep may arise, at least in part, from expectation-driven and attributional processes rather than direct physiological influences.

## Data Availability

The dataset generated during the current study is not publicly available, as data collection is ongoing, but is available from the author upon reasonable request.

## Supplementary Materials

The following supporting information can be downloaded at: https://www.mdpi.com/article/10.3390/clockssleep8020036/s1, Figure S1: Word cloud showing the most commonly reported reasons for ruminating before sleep.
