# Supplementary figures and images for: Who Blames the Moon for Poor Sleep? An Exploratory Online Survey
Source: Clocks Sleep. 2026 Jun 22;8(2):36. doi: 10.3390/clockssleep8020036 (PMC13298107; doi:10.3390/clockssleep8020036)

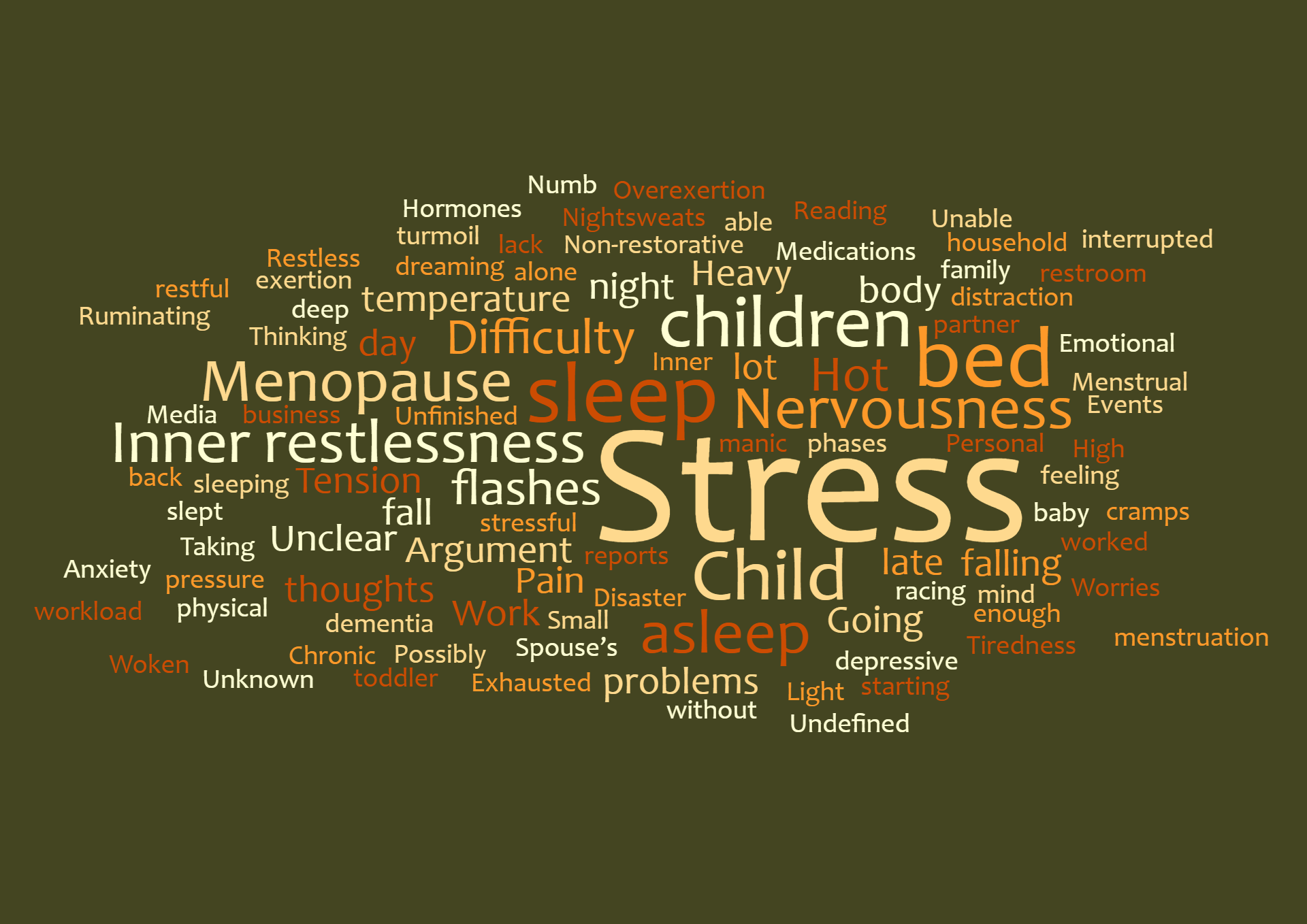

Supplement: Supplementary file 1 [file clockssleep-08-00036-s001.zip › clockssleep-4328376-supplementary.png]
